# Nomogram for Predicting Postoperative Blood Pressure Reduction in Hypertensive Patients: A Single-Center Retrospective Study

**DOI:** 10.7150/ijms.112777

**Published:** 2025-07-28

**Authors:** Zongsu Zhang, Xiaocheng Ma, Zhaochen Li, Haotian Wei, Kaipeng Jia, Chenglong Xu, Shimiao Zhu, Simeng Wen, Changyi Quan

**Affiliations:** 1 Department of Urology, The Second Hospital of Tianjin Medical University, Tianjin, China.; 2 Department of Urology, The Affiliated Hospital of Qingdao University, Qingdao, Shandong, China.

**Keywords:** hypertension, renal tumor, nomogram, prediction model, surgery

## Abstract

**Background:** Hypertension is a major public health problem. In clinical practice, we have observed that when hypertensive patients undergo robotic-assisted partial nephrectomy (RAPN), approximately half of them experience a normalization of their blood pressure (BP) shortly after the surgery. This study aims to investigate the effect of RAPN on BP in hypertensive patients with renal tumor by disassociating nerve tissue around the renal artery.

**Methods:** We reviewed patients with renal tumor requiring RAPN who were admitted to our department from January 2021 to January 2024, with a minimum follow-up of 3 months. A total of 260 hypertensive patients combined with renal tumor were followed up. Univariate and multivariate logistic regression analyses were sequentially employed to determine the factors associated with blood pressure normalization following RAPN. Finally, a nomogram model based on independent risk factors was established and validated.

**Results:** A total of 55.38% (144/260) hypertensive patients combined with renal tumor have achieved blood pressure normalization following RAPN. A multivariate logistic regression analysis showed that preoperative BP (OR=0.145; 95%CI:0.052-0.421; p<0.001), circulatory diseases(OR=15.661; 95%CI:8.611-30.576; p<0.001), plasma renin activity ratio(PRA) (OR=0.071; 95%CI:0.035-0.131; p<0.001), preoperative angiotensin II (AT II) (OR=0.693; 95%CI:0.551-0.861; p=0.002), Body Mass Index (BMI) (OR=0.526; 95%CI:0.355-0.713; p=0.031) were independently correlated with blood pressure normalization. We constructed a nomogram prediction model based on these independent risk factors. Validation through receiver operating characteristic curve, calibration curves, and decision curve analysis demonstrated a strong correlation between predicted and actual occurrence probabilities.

**Conclusion:** This procedure blocks the renin-angiotensin-aldosterone system by disassociating nerve tissue around the renal artery in hypertensive patients, thereby reducing their BP. This surgical method may become a potential new treatment for hypertension in the future.

## Introduction

Approximately 60% of patients with renal tumor are accompanied by hypertension, which is positively associated with an increased risk of kidney cancer^1^, ^2^. Nephrectomy, partial or radical, is the standard therapeutic way for renal tumor. Hypertension is one of the most common chronic diseases and one of the most important risk factors for cardiovascular disease^3^. According to previous studies, sympathetic hyperexcitability is a fundamental part of the pathogenesis of hypertension^4^, and chronic excitation of the renal sympathetic nerve activates the renin-angiotensin-aldosterone system(RAAS), causing a series of complications such as increased BP^4^, ^5^.

In recent years, catheter-based renal sympathetic denervation has been attempted in the treatment of refractory hypertension(RH)^6^, ^7^. Catheter-based renal sympathetic denervation^8^ is a new approach to the treatment of RH and as the mechanism of RH are delved into more profoundly, this approach is expected to find a wider application scope in the treatment of RH^9^, ^10^. It has been found in clinical practice that intraoperative disassociation of the nerve tissue surrounding the renal artery during RAPN may affect the RAAS in hypertensive patients, causing a drop in BP or to a normal level in some hypertensive patients in the early postoperative period. This provides a new idea for the treatment of hypertension, namely that adequate intraoperative freeing of the renal artery and disassociation of the nerve tissue around the renal artery can decrease BP in hypertensive patients.

The nomogram model is a visualization tool designed to predict and assess disease risk or treatment efficacy. It consolidates multiple independent risk factors into a single graphic, enabling the calculation of an individual's predicted risk or prognosis probability. This study starts from the mechanism by which blocking the RAAS can lower BP, and it aims to determine the factors associated with blood pressure normalization following RAPN and to develop and validate a nomogram for hypertensive patients combined with renal tumor who have undergone RAPN.

## Methods

### Study population

This study was approved by the Ethics Committee of the second hospital of Tianjin Medical University (approval number: ky2021k033) and was conducted in accordance with the Declaration of Helsinki (Sixth Revised Edition, 2008) of 1975. We reviewed 260 hypertensive patients combined with renal tumor admitted to the urology department of the Second Hospital of Tianjin Medical University (Tianjin, China) and underwent RAPN from January 2021 to January 2024. Written informed consent was obtained from all patients for this retrospective study. Inclusion criteria for this study included: age >20 years with renal tumor; history of hypertension on admission, taking antihypertensive medication or not taking antihypertensive medication on admission but with blood pressure >140/90 mmHg; admitted to the urology department of our hospital and operated by the same senior surgeon; normal renal function assessed preoperatively; insignificant paraneoplastic syndrome; underwent RAPN with intraoperative disassociation of the nerve tissue around the renal artery. Exclusion criteria included: previous chronic kidney disease, renal insufficiency; having adrenal tumors like primary aldosteronism; having unilateral or bilateral renal artery stenosis; advanced renal tumor with the significant paraneoplastic syndrome; and patients with secondary hypertension.

To better validate this model, we randomly divided the entire study population into training and validation samples in a 7:3 ratio (**Table 1**).

### Study protocol

**Figure 1:**
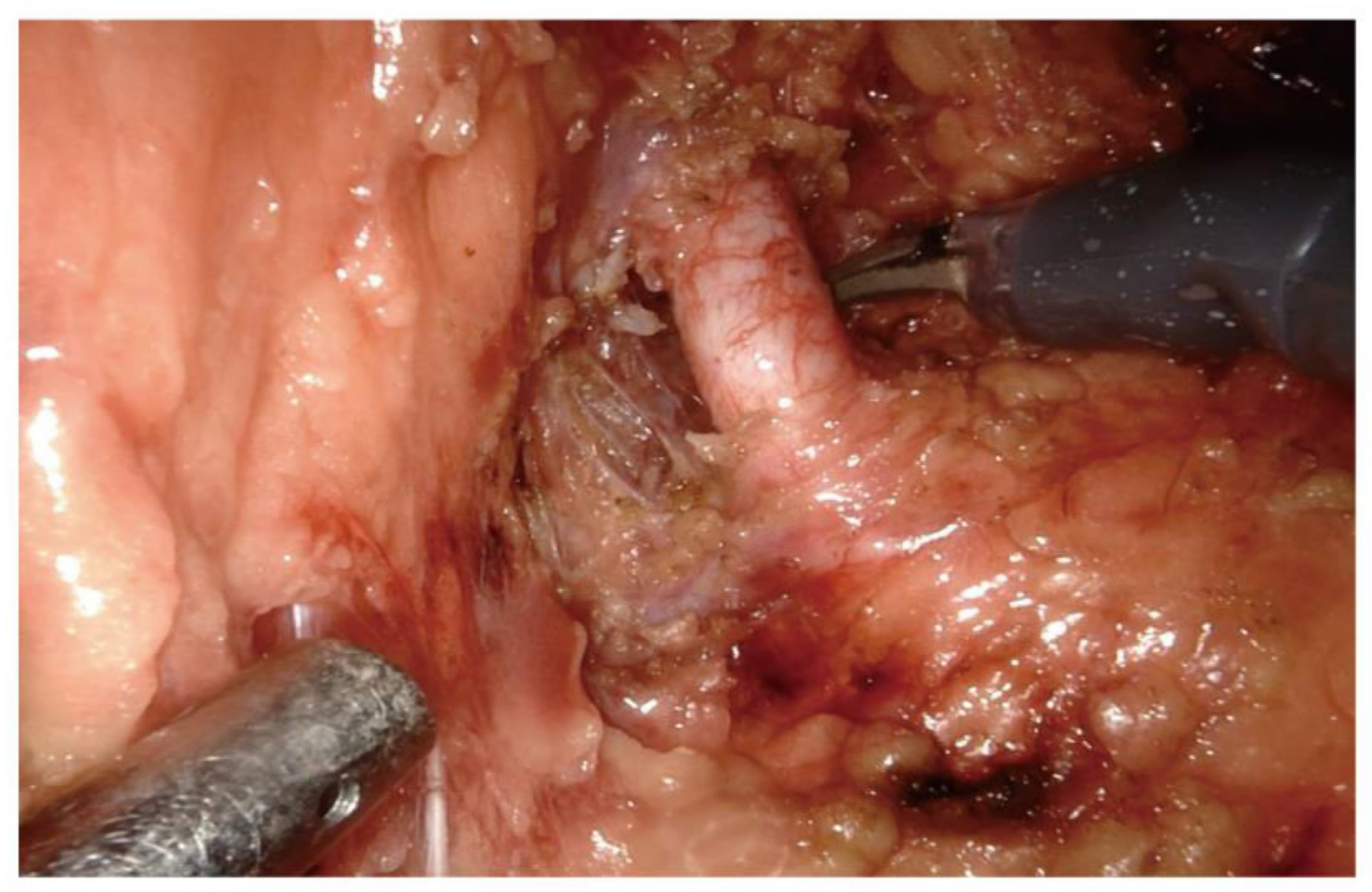
Intraoperative dissection of the nerve tissue around the renal artery during RAPN.

The included patients were all underwent RAPN performed by the same experienced senior surgeon in our department. After placing the patient in the lateral position, general anaesthesia and establishing the surgical space, the perirenal tissue are separated and further separated to the renal hilum, carefully separating the tissue at the hilum, disassociating the nerve tissue and microvessels around the renal artery. Before clamping or dissecting the renal artery, carefully disassociate the nerve tissue around the renal artery so that the surgical field at the renal artery can be clearly exposed to ensure the safe subsequent removal of the renal tumor^11^ (**Figure 1**).

We collected baseline characteristics of all enrolled patients, including age, sex, BMI, current smoking status, diabetes mellitus, history of cardiovascular diseases, tumor laterality, and tumor diameter. We collected these patients' hormone indicators including: ALD, AT II, PRA before and after surgery. Fasting venous blood samples for these hormone indicators were collected on the morning of the second day after admission and analyzed by our institutional clinical laboratory. Comparing whether there were differences in ALD, AT II and PRA values before and after surgery.

BP records were collected from patients during their hospitalization, and office BP measurements were performed by trained and regular health care workers using the same BP measuring device for patients admitted with a history of hypertension. Patients rested for 5 minutes and then BP was measured in each arm. BP measurements were recorded 3 times with a 2-minute interval, recorded in full using a form, and submitted to two regular cardiologists for review. The arm with the higher BP was used for measurement at the follow-up visit. We divided hypertensive patients into two categories according to their preoperative BP control level after taking antihypertensive medication: a. BP <140/90mmhg after medication, b. BP greater than or equal to 140/90mmhg after medication (**Table 1**). Patients were asked to continue their preoperative antihypertensive medication until the day before surgery and to maintain the same type and dose of hypertensive medication or diuretics as before surgery if their postoperative BP was poorly controlled (>140/90 mmHg). If a hypertensive patient's BP is normal (<140/90mmHg) within 3 days after surgery, the medication may be dispensed with and the patient's BP will then continue to be monitored. No change in antihypertensive medication is generally allowed. The patient's compliance with medication is recorded through the patients' diaries. Information relevant to this study was collected when patients were followed up at 3 months postoperatively, including office BP measurements, medication compliance, physical examination, adverse events and other vital signs.

Improvement in BP control level in hypertensive patients was defined as: at 3 months postoperatively, the patient's BP had returned to normal(<140/90mmhg) and required the same or less antihypertensive medication postoperatively. All enrolled patients were categorized into four grades according to the degree of postoperative BP decline. Of these, grade1: postoperative BP better than preoperative BP, not taking antihypertensive medication, maintaining BP <140/90mmhg for at least 3 months postoperatively; grade2: postoperative BP better than preoperative BP, taking less antihypertensive medication, maintaining BP <140/90mmhg for at least 3 months postoperatively; grade3: postoperative BP better than preoperative BP, taking the same antihypertensive medication, maintaining BP <140/90mmhg from surgery to 3 mouths postoperatively; grade4: No significant change in postoperative BP compared to preoperative levels.

Considering that rein in the renal tumor may trigger hypertension, to exclude the effect of decreased hypertension caused by the removal of renin, we randomly selected 9 pairs of renal tumor tissue and normal kidney tissue surrounding the tumor in hypertensive patients who underwent RAPN and measured the concentration of PRA in the tumor tissue and normal kidney tissue by Elisa assay to compare the difference between them. All patients had signed an informed consent. Supernatants made from excised tumor tissue and normal kidney tissue were taken for the Elisa assay according to the instructions of the purchased Human Renin Activity Assay Kit (Jianglai Bio). Three technical replicates of each sample were measured.

### Outcomes

The primary observation indicator was the proportion of blood pressure normalization from baseline to 3 months after surgery in hypertensive patients. Secondary observation indicators were: differences in aldosterone(ALD), angiotensin II(AT II), and plasma renin activity(PRA) before and after surgery; the concentration of PRA measured in renal tumor tissue were generally not higher than in normal kidney tissue in Elisa test; postoperative tumor recurrence and metastasis, comorbidities; any major embolic events leading to end-organ damage^12^; any adverse safety incidents.

### Statistical analysis

Normally distributed continuous variables are expressed as means (standard deviations) and non-normally distributed variables are expressed as medians (interquartile spacing). Categorical data are summarized as frequencies (%). The normality of variable distributions was assessed using the Shapiro-Wilk test. For evaluating the pre- to postoperative changes in blood pressure, we use paired t-tests​​ for within-group comparisons (preoperative vs postoperative systolic BP/ diastolic BP) in normally distributed data and use Wilcoxon signed-rank tests​​ for non-normally distributed changes.

We first performed the univariate logistic regression analysis, using the blood pressure normalization as the dependent variable. Variables with a p-value < 0.05 were included in the multivariate logistic regression model to identify independent risk factors. Linearity between continuous variables and logit(p) was verified by Box-Tidwell test. Multicollinearity was excluded (VIF <2.0) and independence of residuals was confirmed via Durbin-Watson test. We used the Hosmer-Lemeshow test to assess the goodness-of-fit(p>0.05). Interaction terms (e.g., age×diabetes) were tested via Wald test. Known confounders (e.g., BMI, baseline medication) were adjusted in multivariate models. Stratified analysis by variables showed consistent effects. We established a nomogram model based on the independent risk factors determined by multivariate logistic regression analysis. Finally, we assessed the model's discrimination using the ROC curve and validated the model's consistency with a calibration curve. Decision curve analysis (DCA) was used to determine the clinical utility of the nomogram.

Paired t-tests were performed on the measured PRA concentration in 9 pairs of renal tumor tissue versus surrounding normal kidney tissue and the concentration values of blood ALD, AT II, and PRA before and after surgery. Statistical analyses were performed using IBM SPSS 25.0 and R version 4.3.1. Two-tailed P-values <0.05 were considered statistically significant.

## Results

### Baseline characteristics and postoperative complications

Overall, 260 eligible hypertensive patients combined with renal tumor participated in our retrospective study. Average age was 61.75±13.02 years, the followed-up patients included 63 women and 197 men. Patients were on an average of 2.61 antihypertensive medications or diuretics. 250 of the 260 patients were free of complications. Among them, six patients had postoperative pulmonary metastases and was followed up with targeted therapy; four patients had a combination of incisional infections that gradually improved. At 3 months postoperatively, 2 patients had a more significant increase in hypertension after surgery compared with preoperative level due to targeted drug administration. The remaining hypertensive patients did not show a short-term postoperative increase in BP. No major embolic events; no new myocardial infarction, new stroke, coronary and renal artery reintervention, major bleeding, or major vascular complications or other adverse safety events in postoperative patients.

### Postoperative BP changes

**Figure 2:**
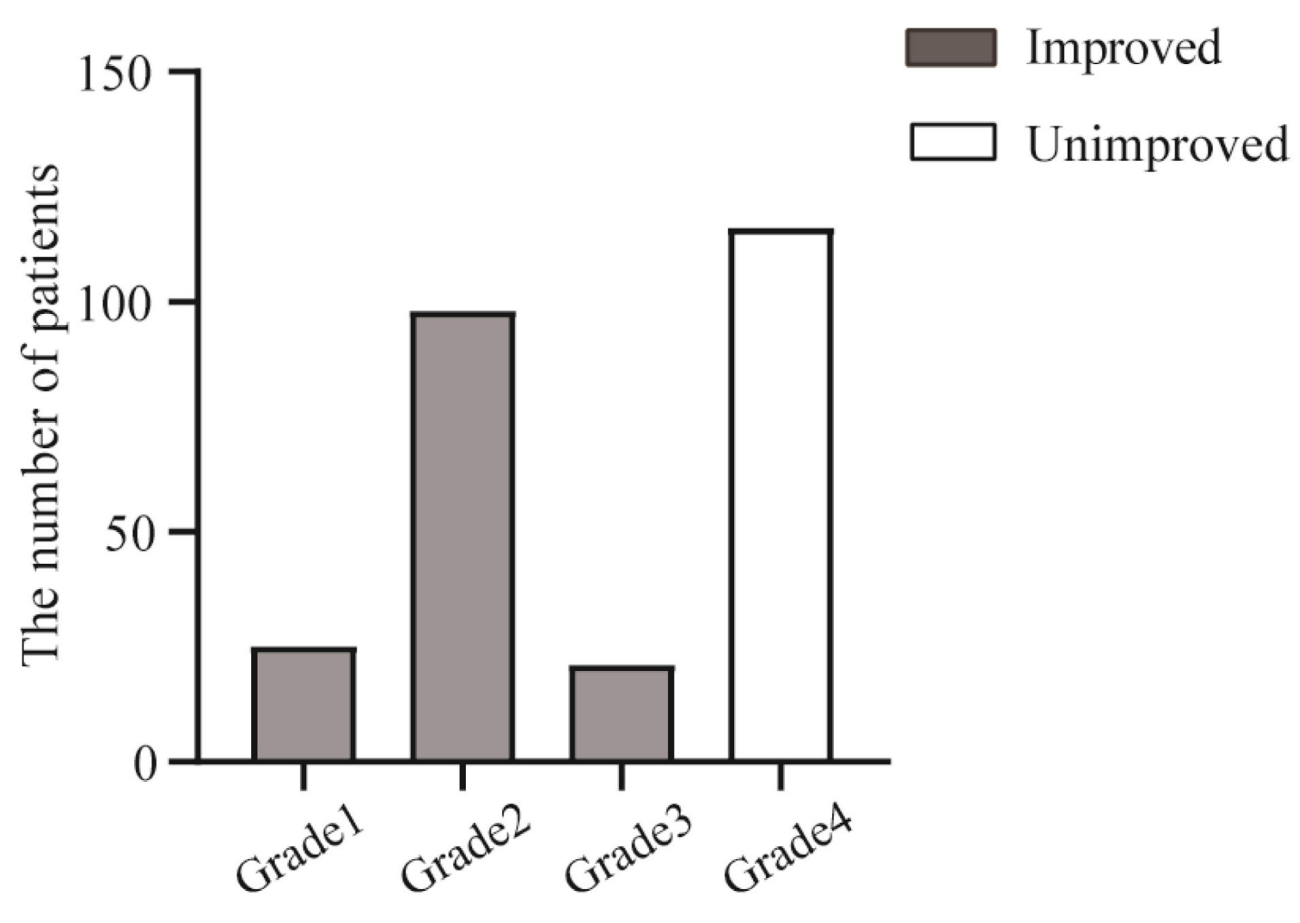
BP grading results at 3-month follow-up postoperatively in enrolled patients (n=260). Grade1: Postoperative BP better than preoperative BP, not taking antihypertensive medication, maintaining BP <140/90mmhg for at least 3 months postoperatively (n=25). Grade2: Postoperative BP better than preoperative BP, taking less antihypertensive medication, maintaining BP <140/90mmhg for at least 3 months postoperatively (n=98). Grade3: Postoperative BP better than preoperative BP, taking the same antihypertensive medication, maintaining BP <140/90mmhg from surgery to 3 mouths postoperatively (n=21). Grade4: No significant change in postoperative BP compared to preoperative levels (n=116).

A total of 55.38% (144/260) hypertensive patients achieved improved blood pressure after surgery (**Figure 2**). Of these, patients with Grade 1-3 were classified as having achieved blood pressure normalization, while those with Grade 4 failed to show blood pressure normalization. Among the patients with improved BP, twenty-five patients required preoperative antihypertensive medication but maintained normal BP (<140/90 mmHg) without medication for 3 days postoperatively and continued without medication until the 3-month follow-up; ninety-eight patients with preoperative BP above 140/90 mmHg maintained normal BP (<140/90 mmHg) with less medication postoperatively, however, brief medication discontinuation led to BP exceeding normal level compared to the first group; twenty-one patients with uncontrolled preoperative BP achieved blood pressure normalization postoperatively and maintained their baseline number of antihypertensive agents, which exceeded that of Grade 2 patients and early postoperative cessation of these medications induced significant BP increases in this population. At the 3-month follow-up, all hypertensive patients demonstrated good medication adherence postoperatively.

### Hormone changes and measurement of PRA concentration

**Figure 3:**
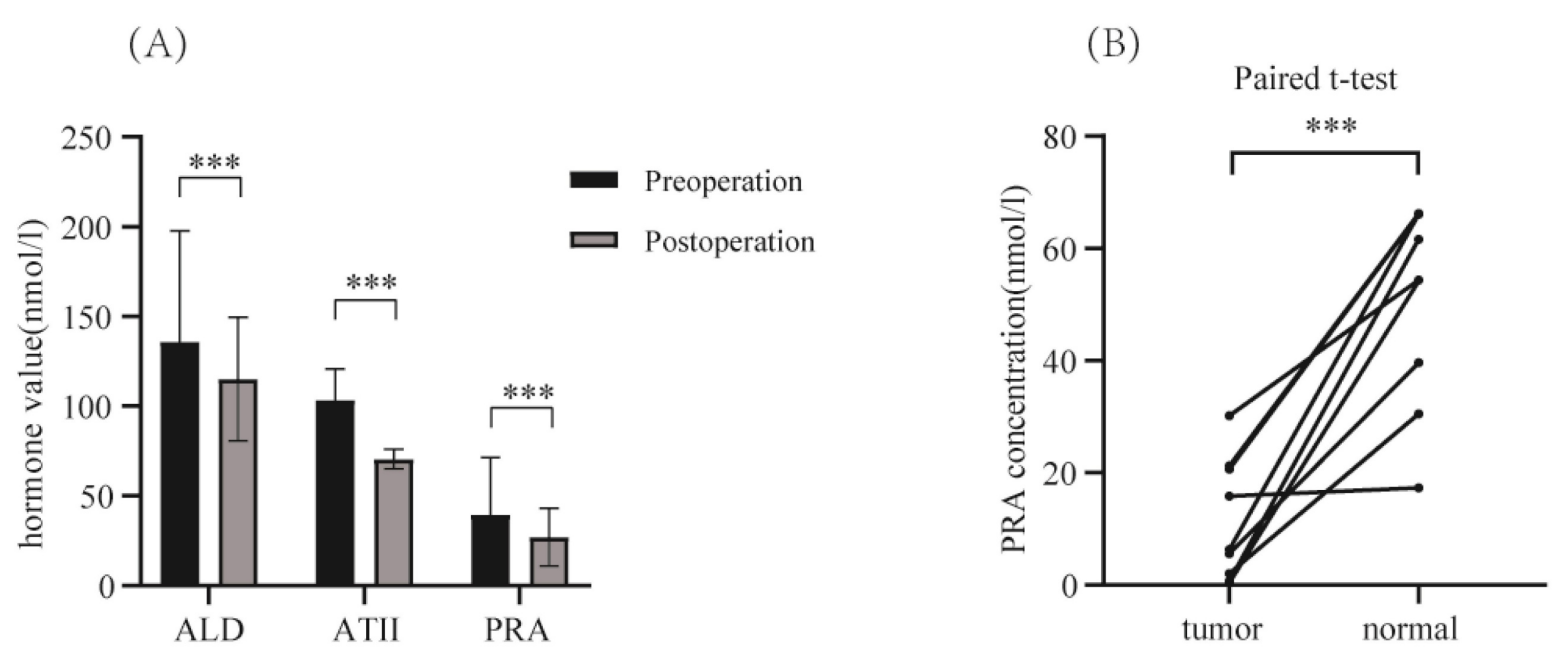
** (A)** Comparison of preoperative and postoperative hormone values (including: ALD, AT II, PRA) in enrolled patients (p<0.001). **(B)** Paired t-test of ELISA results for measured PRA concentration in selected 9 pairs of renal tumor tissue versus normal kidney tissue surrounding the tumor (p<0.001).

We performed paired t-tests to compare preoperative and postoperative hormonal indicators reflecting hypertension for all patients (**Figure 3**). Significant differences were observed in blood ALD, AT II, and PRA values before and after surgery (P<0.05). Additionally, for 9 pairs of renal tumor tissue and normal kidney tissue from 9 hypertensive patients with kidney cancer, we measured PRA concentrations using the Elisa assay, and conducted paired t-tests on the results. The findings revealed that PRA concentration in renal tumor tissue was significantly lower than in the surrounding normal kidney tissue (p<0.001) (**Figure 3**). This suggests that renin in the surgically removed tumor tissue is not the cause of the BP reduction in hypertensive patients.

### Factors associated with postoperative decrease in BP

**Figure 4:**
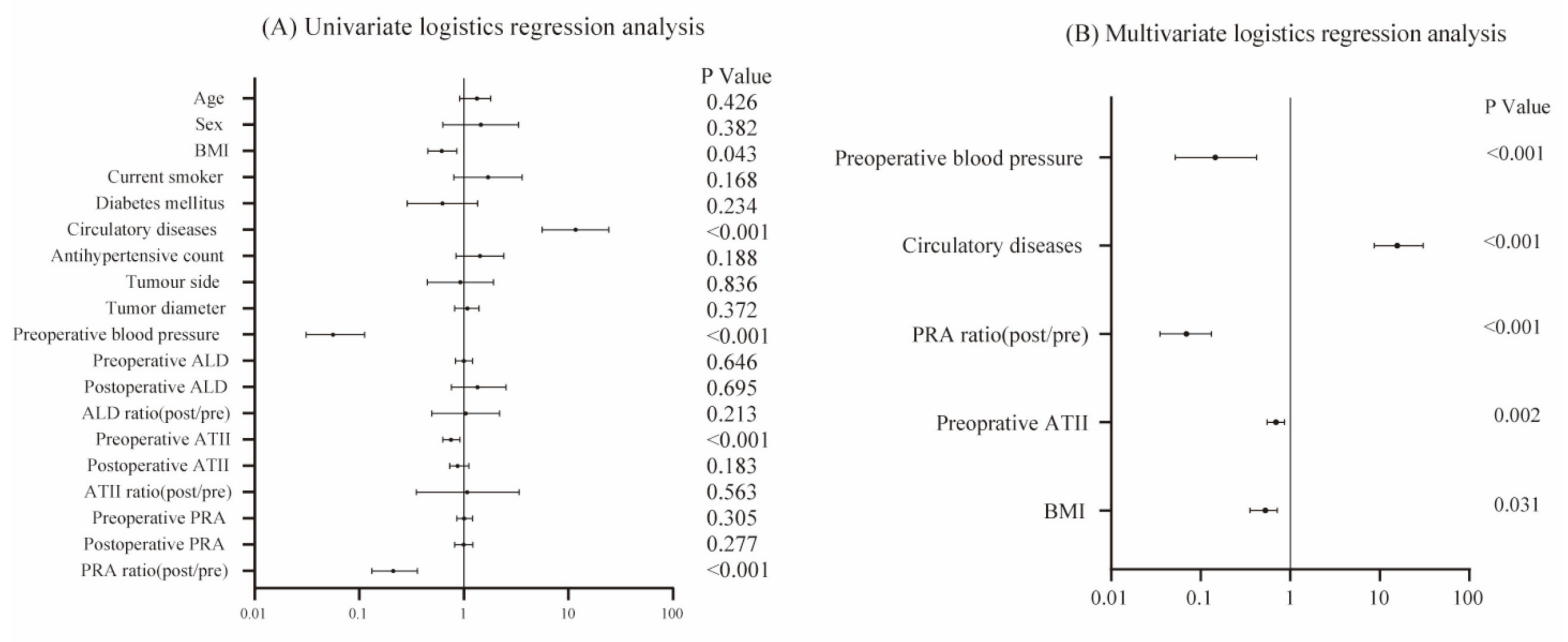
Forest plot of univariate (A) and multivariate (B) logistic regression analyses. In the multivariate model, five independent risk factors associated with blood pressure normalization were identified, including: BMI (OR=0.526, CI=0.355-0.713, p=0.031), postoperative/preoperative PRA ratio (OR=0.071, CI=0.035-0.131, p<0.001), preoperative AT II (OR=0.693, CI=0.551-0.861, p=0.002), circulatory diseases (OR=15.661, CI=8.611-30.576, p<0.001) and preoperative BP (OR=0.145, CI= 0.052-0.421, p<0.001).

To further investigate the causes of decreased BP in hypertensive patients after RAPN, we conducted univariate and multivariate logistic regression analyses, using the blood pressure normalization as the dependent variable. The univariate logistic regression analysis revealed that improvement in hypertension was significantly associated with BMI (p=0.043), postoperative/preoperative PRA ratio (p<0.001), preoperative AT II (p<0.001), circulatory diseases (p<0.001), and preoperative BP (p<0.001) (**Table 2 and Figure 4**). No significant associations were found between reduced hypertension and age, sex, diabetes mellitus, current smoker status, tumor side, tumor diameter, or antihypertensive medication count in the univariate analysis. Variables with p<0.05 were included in the multivariate logistic regression model. The multivariate logistic regression analysis showed that the postoperative/preoperative PRA ratio (p<0.001), preoperative AT II (p=0.002), BMI (p=0.031), circulatory diseases (p<0.001), and preoperative BP (p=0.041) were independently associated with the blood pressure normalization (**Table 2 and Figure 4**).

### Development and validation of the nomogram

**Figure 5:**
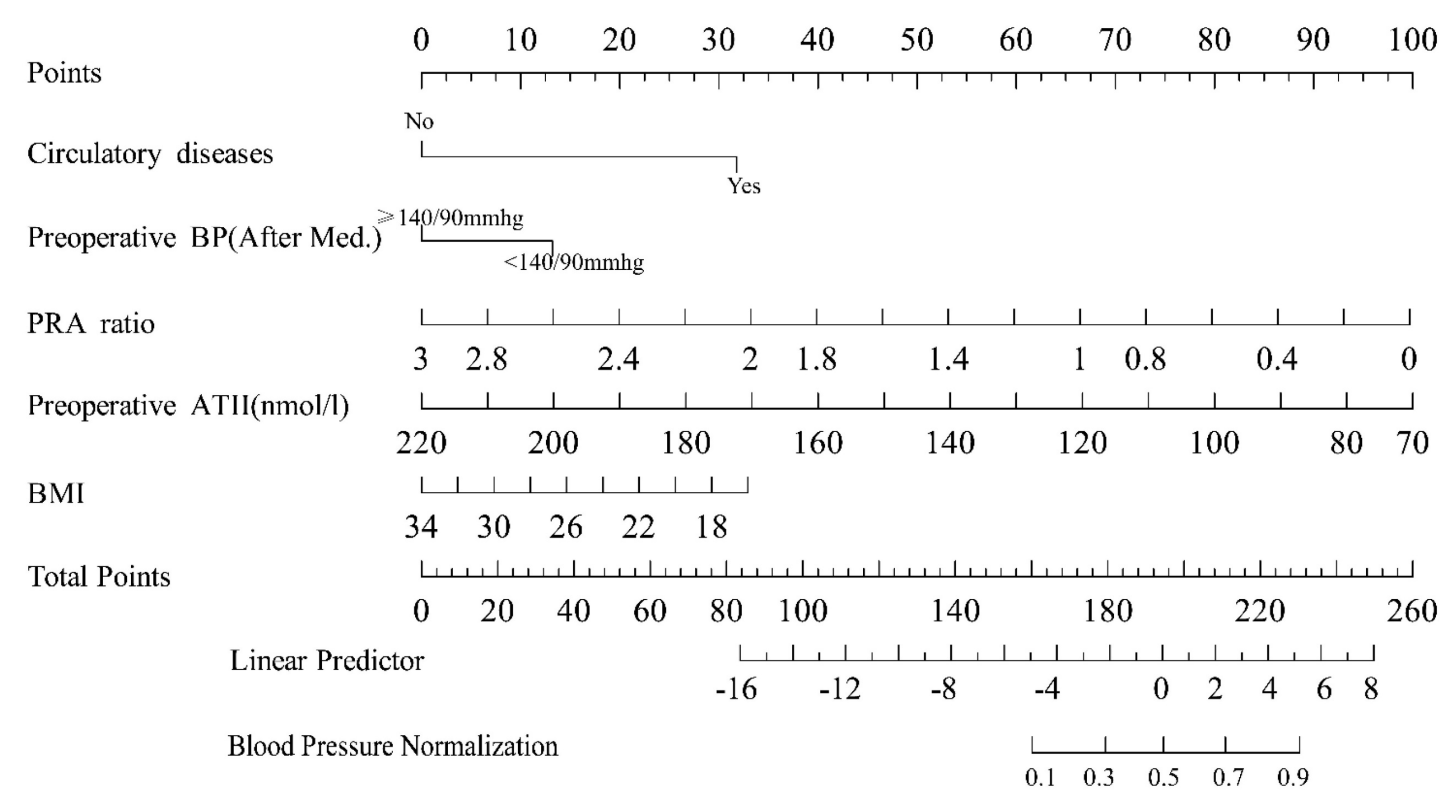
A nomogram model was established based on the independent risk factors identified through multivariate logistic regression analysis. For each patient, the corresponding scores for individual factors were determined by simply drawing a vertical line upwards (e.g. patients with specific circulatory system diseases were assigned 31 points). These scores were then summed up to calculate the total score. After that, the corresponding point on the total score axis was located, and a downward-drawn vertical line was used to predict the likelihood of blood pressure normalization.

Based on five independent predictors identified through multivariable logistic regression analysis, a nomogram model was developed to predict the probability of the blood pressure normalization (**Figure 5**). In the nomogram, the contribution of each predictor is visually represented by the length of its corresponding line segment. Patients were scored based on each risk factor's criteria, and total scores were calculated by summing individual scores. Higher total scores correlated with an increased probability of successful surgical blood pressure control.

**Figure 6:**
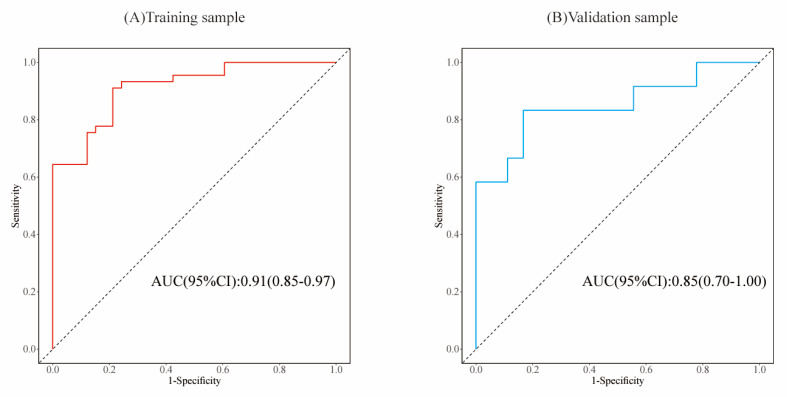
The AUCs of the training set samples (A) and validation set samples (B) indicate that the model exhibits a remarkable discriminatory capacity.

**Figure 7:**
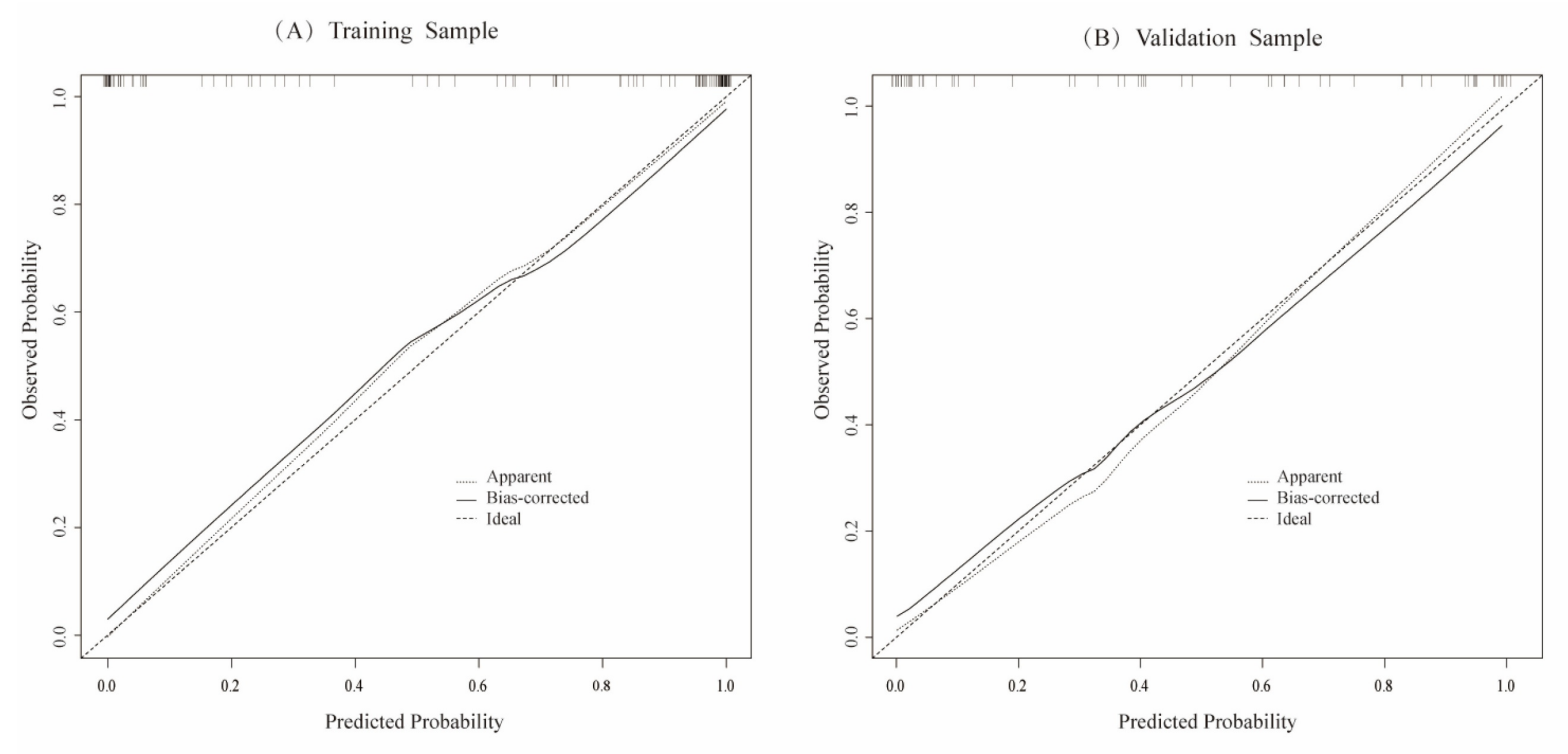
The calibration curves of the training set samples (A) and the validation set samples (B) are used to evaluate the predicted and actual values of the likelihood of blood pressure normalization. The results show that there is a good consistency between the predicted risk assessment and the actual risk assessment.

**Figure 8:**
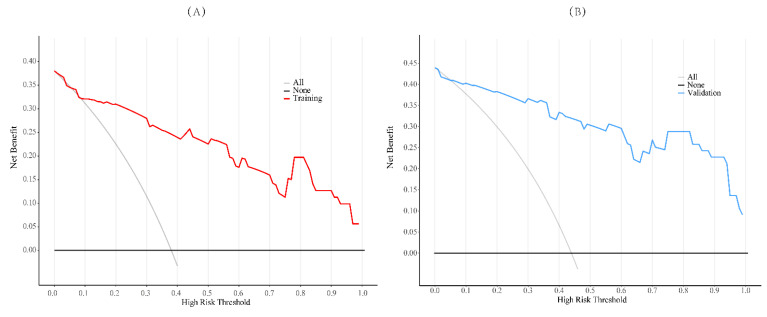
Decision curve analysis was employed to assess the clinical utility of the training set samples (A) and the validation set samples (B). In this analysis, the decision curve yielded a relatively substantial net benefit within the range from 5.0% to 95.0%.

To evaluate the model's discriminative ability, receiver operating characteristic (ROC) curves were plotted, and the area under the curve (AUC) was calculated in both training and validation cohorts (**Figure 6**). The model demonstrated good discrimination, with the training set achieving an AUC of 0.91 and the validation set 0.85. Calibration curves confirmed consistency between predicted probabilities and observed outcomes in both cohorts (**Figure 7**). Furthermore, decision curve analysis (DCA) indicated a positive net benefit difference across threshold probabilities ranging from 0.05 to 0.95, supporting the model's clinical utility (**Figure 8**).

## Discussion

Our study demonstrates that laparoscopic dissection of the nerve tissue surrounding the renal artery effectively reduces BP in hypertensive patients, with sustained effects lasting for at least three months. Multivariate analysis identified postoperative/preoperative plasma renin activity (PRA) ratio, circulatory diseases, preoperative AT II levels, BMI, and baseline BP as independent predictors of blood pressure normalization (p<0.05). Importantly, no major adverse safety events were associated with this procedure. Four postoperative wound infections were resolved with antibiotics and dressing changes. Six distant metastases were treated with targeted therapies, preventing further progression. These postoperative infections and distant metastases were not related to the dissection of nerve tissue around the renal artery in our procedure.

Previous studies on catheter-based renal sympathetic denervation (RDN)^6^, ^8^ failed to identify which subgroups of refractory hypertensive patients would benefit most from this technique. Moreover, the procedure requires fluoroscopic guidance via femoral artery access to reach the renal artery, carrying inherent complications and facing challenges in clinical adoption. In contrast, our study has identified specific preoperative characteristics that predict better blood pressure improvement after laparoscopic disassociation of the nerve tissue around the renal artery. Hypertension is known to cause microvascular thinning and inadequate perfusion of peripheral tissues, potentially leading to tissue damage and end-organ injury^13^. It is a well-established risk factor for peripheral vascular disease, with baseline BP strongly associated with the development of peripheral arterial disease^14^, ^15^. Hypertension is a major risk factor for all clinical manifestations of coronary artery disease, as well as for death and ischemic events following acute coronary syndrome^16^. Our study demonstrated that hypertensive patients with more complex underlying circulatory diseases exhibited greater BP reduction after the procedure, underscoring a pathophysiological link between circulatory dysfunction and hypertension severity. Moreover, better baseline BP control predicted superior outcomes, suggesting this intervention complements rather than replaces conventional high BP management. The paired t-test indicated that postoperative PRA was significantly lower in enrolled patients compared to the preoperative period. Measurement of PRA can be used to help guide antihypertensive drug therapy, which is clinically feasible and can be easily integrated into modern clinical practice^17^, ^18^. PRA is also a marker of cardiovascular risk in hypertensive patients and is directly related to cardiovascular morbidity and mortality in hypertensive patients^19^, ^20^. Extensive evidence supports the concept that high renin levels predict future cardiovascular disease and death, particularly in hypertensive patients^21^. Our procedure, which involves dissecting nerve tissue around the renal artery, may block the RAAS and reduce PRA concentration, potentially becoming an important method to decrease BP and highlighting PRA as a valid predictor of hypertension risk. Our Elisa results showed significantly lower PRA concentrations in renal tumor tissue compared to normal kidney tissue surrounding the tumor, suggesting that renin removal from the renal tumor may not be a major factor in BP reduction in hypertensive patients. Dysregulation of AT II signaling is widely recognized as a pivotal mechanism driving increased sympathetic excitation in hypertension^22^, ^23^. AT II plays a significant role in regulating the cardiovascular system, and activating the sympathetic nervous system, contributing to hypertension and cardiovascular disease^24^. Elevated blood pressure in obese individuals is often associated with activation of the systemic RAAS and increased AT II levels^25^, ^26^. The elevated plasma AT II levels may result from adipose tissue's enhanced capacity to produce AT II through increased angiotensinogen^25^, ^27^. These results reflect obesity-related RAAS activation patterns.

Lastly, this study has several limitations. First, although we made efforts to increase the sample size, it remains relatively small; future multi-center studies with larger cohorts and extended follow-up durations are required for validation. Second, the single-center design may limit generalizability, as perioperative management protocols in this study could differ from standard practices at other institutions. Furthermore, while internal validation was performed, external validation across independent datasets has not been conducted. Moreover, although the decision curve analysis demonstrated favorable clinical utility across a threshold probability range of 5% to 95%, its generalizability may be limited by the single-center cohort and moderate sample size. Therefore, future investigations should focus on expanding sample sizes and incorporating multi-center cohorts to strengthen the reliability of these findings.

## Conclusion

In conclusion, this study developed a nomogram prediction model to forecast the probability of blood pressure normalization in hypertensive patients after laparoscopic adequately dissection of the nerve tissue around the renal artery. This model offers a new surgical approach for treating hypertension in such hypertensive patients particularly for patients with circulatory comorbidities and controlled baseline BP and helps doctors counsel and treat hypertensive patients with potential risks. Further research is needed to validate this with more evidence.

## Figures and Tables

**Table 1:** Baseline clinical characteristics of enrolled patients.

Variable	Training sample(n=182)	Validation sample(n=78)
Age	61.90±6.23	61.42±6.58
Gender		
Male	138(75.82%)	59(75.64%)
Female	44(24.18%)	19(25.36%)
BMI (kg/m^2^)	26.79±3.72	26.70±3.87
Current smoker	84(46.15%)	34(43.59%)
Diabetes mellitus	75(41.20%)	36(46.15%)
Circulatory diseases	118(64.84%)	50(64.10%)
Antihypertensive count	2.63±0.71	2.55±0.66
Tumor side		
Left	101(55.49%)	44(56.41%)
Right	81(44.51%)	34(43.59%)
Tumor diameter	4.20±1.80	3.66±1.72
Preoperative blood pressure		
<140/90mmhg	106(58.24%)	45(57.69%)
≥140/90mmhg	76(41.76%)	33(42.31%)
Preoperative ALD (nmol/l)	136.33±64.20	134.82±61.88
Postoperative ALD (nmol/l)	119.43±35.72	104.77±22.73
ALD ratio(post/pre)	0.90±0.39	0.86±0.26
Preoperative AT II (nmol/l)	103.19±14.10	104.20±19.48
Postoperative AT II (nmol/l)	70.07±8.33	71.49±5.93
AT II ratio(post/pre)	0.69±0.10	0.70±0.10
Preoperative PRA (nmol/l)	43.36±32.23	30.50±29.00
Postoperative PRA (nmol/l)	29.16±16.32	22.05±16.09
PRA ratio(post/pre)	0.83±0.60	0.85±0.74

Data are mean (SD), median (range)or number (%); BMI=Body mass index; ALD= aldosterone; AT II =angiotensin II; PRA=plasma renin activity.

**Table 2:** Identifying risk factors influencing surgical efficacy: a univariate and multivariate logistic regression analysis.

Factor	Univariable analysis			Multivariable analysis		
	OR	95%CI	p value	OR	95%CI	p value
Age	1.332	0.912-1.811	0.426			NI*
Sex, n (%)						NI
Female	Ref	Ref	Ref			
Male	1.449	0.631-3.329	0.382			
BMI (kg/m^2^)	0.612	0.451-0.856	0.043	0.526	0.355-0.713	0.031
Current smoker						NI
Yes	1.701	0.800-3.611	0.168			
No	Ref	Ref	Ref			
Diabetes mellitus						NI
Yes	0.624	0.287-1.356	0.234			
No	Ref	Ref	Ref			
Circulatory diseases				15.661	8.611-30.576	<0.001
Yes	11.691	5.602-24.381	<0.001			
No	Ref	Ref	Ref			
Antihypertensive count	1.424	0.841-2.412	0.188			NI
Tumor side						NI
Left	Ref	Ref	Ref			
Right	0.926	0.447-1.916	0.836			
Tumor diameter(cm)	1.082	0.815-1.395	0.372			NI
Preoperative blood pressure(mmhg)				0.145	0.052-0.421	<0.001
<140/90mmhg	Ref	Ref	Ref			
≥140/90mmhg	0.056	0.031-0.112	<0.001			
Preoperative ALD (nmol/l)	0.999	0.833-1.211	0.646			NI
Postoperative ALD (nmol/l)	1.351	0.763-2.532	0.695			NI
ALD ratio(post/pre)	1.042	0.494-2.199	0.213			NI
Preoperative AT II (nmol/l)	0.753	0.631-0.921	<0.001	0.693	0.551-0.861	0.002
Postoperative AT II (nmol/l)	0.871	0.731-1.115	0.183			NI
AT II ratio(post/pre)	1.076	0.351-3.376	0.563			NI
Preoperative PRA (nmol/l)	1.005	0.856-1.211	0.305			NI
Postoperative PRA (nmol/l)	0.996	0.815-1.213	0.277			NI
PRA ratio(post/pre)	0.211	0.132-0.361	<0.001	0.071	0.035-0.131	<0.001

*:NI: Not include.
